# Tetrandrine slows disease progression on high-resolution computed tomography and lung function decline in artificial stone-associated silicosis: a retrospective cohort study

**DOI:** 10.1186/s12890-025-03821-8

**Published:** 2025-07-19

**Authors:** Shaowei Zhou, Jin Shi, Zidan Chen, Luqin Bian, Limin Huang, Ling Mao

**Affiliations:** Department of Pneumoconiosis, Shanghai Pulmonary Hospital, Tongji University, Shanghai, 200433 China

**Keywords:** Antifibrotic agents, Silicosis, Benzylisoquinolines, Computed tomography, Pulmonary function tests

## Abstract

**Background:**

Silicosis, a progressive fibrotic lung disease caused by silica dust inhalation, is a significant occupational health concern, particularly among artificial quartz stone workers. Tetrandrine, a bisbenzylisoquinoline alkaloid, is the only plant-derived drug approved for the treatment of silicosis in China. The present study aimed to evaluate the efficacy of tetrandrine in slowing the progression of artificial stone-associated silicosis.

**Methods:**

In this retrospective cohort study, patients were divided into an observation group (*n* = 53), which received tetrandrine (60 mg, 3 times daily for 6 consecutive days, followed by a 1-day break, with each cycle lasting 3 months), and a control group (*n* = 26), which received only symptomatic treatment. High-resolution computed tomography (HRCT) and pulmonary function tests (PFTs) were performed at baseline and after 12 months of treatment. Progression, stability, or improvement in HRCT findings and changes in PFT parameters were analyzed. Continuous variables and categorical variables were analyzed using the *t*-test and chi-square test, respectively, for statistical comparisons.

**Results:**

After 12 months, 49.1% of patients in the observation group exhibited improvement in HRCT findings, compared to none in the control group, in which 84.6% of individuals exhibited progression. PFT findings improved in the observation group, whereas they significantly declined in the control group (*p* < 0.001). Patients treated with tetrandrine for more than 6 months experienced greater improvements in HRCT findings and pulmonary function than those in the control group. Adverse reactions to tetrandrine, including facial pigmentation and liver function abnormalities, were mild.

**Conclusions:**

Tetrandrine significantly mitigated HRCT-detected disease progression and lung function decline in patients with artificial stone-associated silicosis, particularly after prolonged treatment. These findings suggest that tetrandrine may serve as a viable therapeutic option for managing artificial stone-associated silicosis.

## Background

Silicosis is a progressive fibrotic lung disease caused by prolonged inhalation of free silica dust. Even after dust exposure ceases, lung tissue structure and function continue to deteriorate, significantly affecting patient quality of life and potentially leading to fatal outcomes [1, 2]. The inhalation of respirable crystalline silica (RCS) dust is a causative factor in the pathogenesis of silicosis [3, 4]. Unfortunately, conclusive evidence that silica-induced lung fibrosis is reversible is lacking, and, to date, no effective treatment has been established for silicosis [5, 6].

Artificial stone is an engineered material composed of small rock particles rich in silica bound together with polymer resin. To achieve the desired appearance, additives such as colored glass, shells, and metals may be incorporated before curing at high temperatures [7, 8]. Unlike natural stones, artificial stones contain up to 90% silica [9, 10]. Moreover, cutting and grinding artificial stones generate large amounts of RCS, making wet processing insufficient for effectively controlling dust inhalation [11]. Notably, a dose-response relationship exists between RCS exposure and abnormalities in pulmonary function and high-resolution computed tomography (HRCT) findings [12]. In recent years, a growing number of silicosis cases have been reported among artificial stone workers, with the disease exhibiting a more severe and rapidly progressing course than natural stone-associated silicosis, making this a critical occupational health concern [13].

Tetrandrine is a bisbenzylisoquinoline alkaloid extracted from the roots of *Stephania tetrandra* [14]. It is currently the only plant-derived drug approved for the treatment of silicosis in China [15, 16]. Unlike silicosis caused by natural stone, patients with artificial stone-related silicosis experience a more rapid decline in lung function and worsening imaging findings, highlighting the need for further investigations into the therapeutic effects of tetrandrine in these patients.

Therefore, this study retrospectively analyzed the clinical data of patients with artificial stone-related silicosis, aiming to evaluate the efficacy of tetrandrine and assess the impact of long-term medication use on chest HRCT findings and lung function.

## Patients and methods

### Ethics

This study was conducted in accordance with the ethical standards of Shanghai Pulmonary Hospital and the Declaration of Helsinki and was approved by the Institutional Review Board of Shanghai Pulmonary Hospital (Approval Number: K22-313). Written informed consent was obtained from all participants.

### Study design and population

This retrospective cohort study was designed according to the Strengthening the Reporting of Observational Studies in Epidemiology (STROBE) statement. Patients with artificial stone-related silicosis who were hospitalized in the Pneumoconiosis Department of Shanghai Pulmonary Hospital between October 2017 and October 2023 were enrolled in this retrospective cohort study. The observation group received tetrandrine treatment for at least 3 months, while the control group did not receive any tetrandrine. The inclusion criteria were as follows: (1) age 18–70 years, with no sex restrictions; (2) exposure to artificial stone dust while cutting, grinding, and drilling artificial stone slabs, with dust exposure ceased by the time of enrollment in this study; (3) diagnosis of silicosis according to the Chinese Occupational Pneumoconiosis Diagnostic Criteria (GBZ70-2015); and (4) lung function tests and CT conducted at the time of enrollment and both repeated 12 ± 3 months later. Exclusion criteria were as follows: (1) known comorbidities, including respiratory infections, pneumothorax, pleural effusion, active tuberculosis, nontuberculous mycobacteriosis, lung tumors, or other interstitial lung diseases; (2) other organ dysfunctions that could affect pulmonary function or HRCT findings; (3) prior use of antifibrotic medications, such as pirfenidone and nintedanib; and (4) participation in other clinical trials that could influence the results of this study. The stage of silicosis was determined based on perfusion, distribution of small opacities, and presence of progressive massive fibrosis (PMF).

### Treatment protocol

Based on their clinical presentation, all patients received symptomatic treatment, including inhaled or oral bronchodilators, oxygen therapy, antitussives, expectorants, and hepatoprotective drugs. The observation group additionally received tetrandrine 60 mg per dose, 3 times daily for 6 consecutive days, followed by a 1-day break. Each treatment cycle lasted 3 months, followed by a 1-month break, and then repeated.

### HRCT assessment

HRCT images, including baseline and follow-up scans, were retrieved from the picture archiving and communication system of Shanghai Pulmonary Hospital. All images were independently reevaluated by two experienced radiologists in reading pneumoconiosis radiographs who were blinded to the patient groups. The following radiographic changes were assessed according to the reported silicosis HRCT findings: small nodular opacities, ground-glass opacities (GGO), semiconfluent small opacities, PMF, reticulonodular opacities, and patchy consolidation [17, 18]. Progression was defined as occurrence of any of the following changes compared with baseline: (1) increase in small nodular opacities, (2) appearance or increase in diffuse GGO; (3) appearance or enlargement of the PMF, (4) increase in reticulonodular opacities, and (5) increase or enlargement of patchy consolidation. Improvement was defined as occurrence of any of the following changes compared with baseline, with no aggravation of other image features: (1) reduction in small nodular opacities, (2) reduction in GGO, (3) shrinkage of PMF, (4) reduction in reticulonodular opacities, and (5) reduction or shrinkage of patchy consolidation. If no significant changes were observed between baseline and follow-up scans, HRCT findings were classified as stable.

### Pulmonary function tests (PFTs)

PFTs were conducted at the Pulmonary Function Laboratory of Shanghai Pulmonary Hospital using the MasterScreen spirometry system (Jaeger, Germany). Baseline and follow-up results were recorded, and the following parameters were assessed: forced vital capacity (FVC), percentage of the predicted value (FVC%), forced expiratory volume in one second (FEV_1_) and its percentage of the predicted value (FEV_1_%), diffusing capacity for carbon monoxide as a percentage of the predicted value (DLCO%). Predicted values were calculated using reference equations established for Chinese adults in 1988. All test results were obtained from the medical records of the patients.

### Basic characteristics of patients

Clinical data, including age, sex, height, weight, smoking history, occupational history (time of initial dust exposure and time of cessation), and adverse reactions related to tetrandrine, were collected from inpatient and outpatient medical records. The classification and grading of adverse reactions were as follows: alanine aminotransferase or aspartate aminotransferase levels not exceeding 3 times the upper limit of normal, skin itching or diarrhea within one week, work-compatible drowsiness, fatigue, or decreased appetite, and facial pigmentation were all considered mild adverse reactions.

### Statistical analysis

Statistical analyses were performed using SPSS 24.0 software (IBM Corp., Armonk, NY, USA). Continuous variables are expressed as mean ± standard deviation ($\:\stackrel{-}{X}$± s), and comparisons between groups were conducted using *t*-tests. Paired *t*-tests were used for within-group comparisons before and after the treatment. Categorical variables are analyzed using the chi-square (χ²) tests or Fisher’s exact test, as appropriate. Based on clinical experience, researchers expected that the disease stage before treatment and the duration of treatment may have a significant impact on therapeutic outcomes in patients with silicosis. Therefore, a binary logistic regression analysis was conducted to evaluate the effects of baseline disease stage and treatment duration on HRCT improvement. Statistical significance was set at *p* < 0.05.

## Results

### Baseline characteristics

**Fig. 1:**
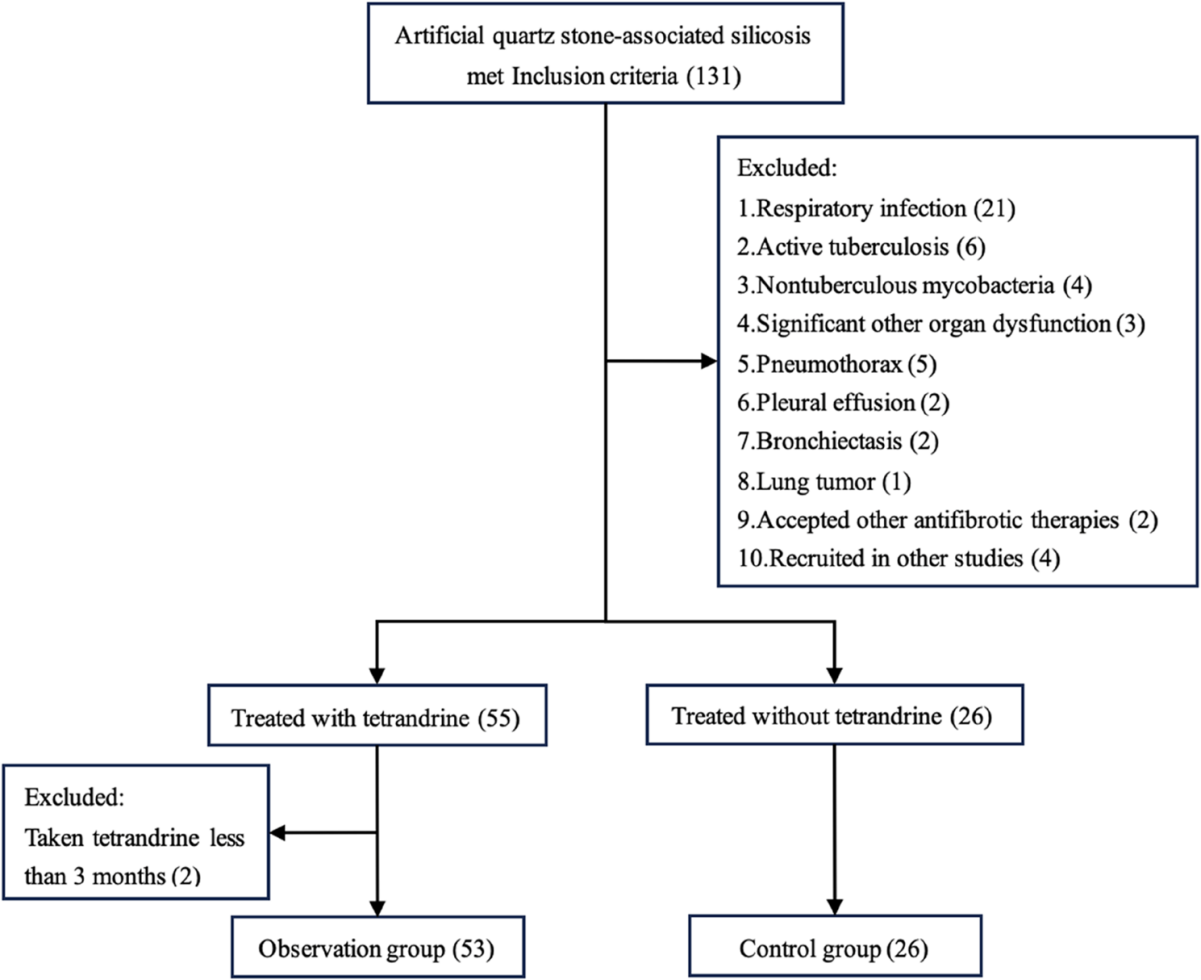
Study flowchart of participants

A total of 131 patients met the inclusion criteria. After applying the exclusion criteria, the observation group consisted of 53 patients (mean age: 40.0 ± 7.7 years), while the control group included 26 patients (mean age: 40.7 ± 10.3 years) (Fig. 1). No significant differences were noted between the two groups in terms of baseline characteristics, including sex, age, body mass index (BMI), smoking history, duration of dust exposure, and silicosis stage (Table 1). Similarly, no significant difference was observed in the incidence of small nodular opacities, GGO, semiconfluent small opacities, PMF, reticulonodular opacities, or patchy consolidations between the two groups at baseline. The lung function indices of the two groups were comparable.

**Table 1:** Characteristics of the observation and control groups

Characteristics		Observation	Control	χ^2^/t	*p*-value
Male, n (%)		52 (98.1)	25 (96.2)	0.000	1.000
Age at enrollment (years)		40.7 ± 10.3	40.0 ± 7.7	0.302	0.764
BMI (kg/ss)		23.8 ± 3.4	23.0 ± 3.9	0.910	0.366
Smoker, n (%)		30 (56.6)	15 (57.7)	0.008	0.927
Dust exposure time (years)		6.5 ± 2.6	6.7 ± 3.0	−0.284	0.777
Time from first exposure (years)		8.8 ± 3.0	8.0 ± 2.6	1.153	0.252
Stage of silicosis, n (%)	I	8 (15.1)	7 (26.9)	1.610	0.447
	II	18 (34.0)	8 (30.8)		
	III	27 (50.9)	11 (42.3)		
Small nodular opacities, n (%)		49 (92.5)	24 (92.3)	0.000	1.000
GGO, n (%)		27 (50.9)	13 (50.0)	0.006	0.937
Semiconfluent of small opacities, n (%)		10 (18.9)	4 (15.4)	0.005	0.946
PMF, n (%)		17 (32.1)	7 (26.9)	0.219	0.640
Reticulonodular opacities, n (%)		44 (83.0)	20 (76.9)	0.118	0.731
Patchy consolidations, n (%)		32 (60.4)	19 (73.1)	1.229	0.268
FVC (mL)		2933.0 ± 852.4	3033.9 ± 896.7	−0.486	0.629
FVC%		71.6 ± 17.8	73.6 ± 21.2	−0.434	0.666
FEV_1_ (mL)		2263.2 ± 845.6	2440.4 ± 769.9	−0.900	0.371
FEV_1_%		66.7 ± 22.2	71.9 ± 23.2	−0.970	0.335
DLCO%		76.2 ± 20.2	82.7 ± 22.2	−1.259	0.212

*BMI* Body mass index, *DLCO%* Diffusing capacity for carbon monoxide as a percentage of the predicted value, *FEV*_*1*_ Forced expiratory volume in one second, FEV_1_% percentage of the predicted FEV_1_, *FVC* Forced vital capacity, *FVC%* Percentage of the predicted FVC, *GGO* Ground-glass opacities, *PMF* Progressive massive fibrosis

### Chest HRCT findings

After the 12-month observation period, none of the patients in the control group exhibited improvement, whereas 22 (84.6%) exhibited disease progression on HRCT images. In the observation group, 26 patients (49.1%) showed improvement, while nine (17.0%) exhibited disease progression on HRCT images. The difference between the two groups was statistically significant (*p* = 0.000). In the observation group, 30 patients had taken tetrandrine for more than 6 months, while 23 received it for 6 months or less. In the two subgroups classified based on the time of tetrandrine use, 19 patients (63.3%) exhibited improvement in the > 6 months subgroup, which was significantly higher than the 7 patients (30.4%) demonstrating improvement in the ≤ 6 months subgroup (*p* = 0.005).

In the binary logistic regression analysis, both variables (baseline staging and treatment duration) were significant, and the Hosmer–Lemeshow goodness-of-fit test indicated that the model adequately fit the data (*p* = 0.225). The model was statistically significant (*p* = 0.000), with Nagelkerke’s R² = 0.560. Baseline staging revealed a negative impact on HRCT improvement (Odds ratio [OR] 0.4, 95% confidence interval [CI] 0.1–0.9), while treatment duration was associated with HRCT improvement (OR 1.7, 95% CI 1.3–2.2). These results suggest that both baseline staging and treatment duration significantly influenced improvement assessed via HRCT, highlighting the positive impact of early intervention and prolonged treatment.

In the observation group, 19 patients (70.4%) with GGO, 16 (50.0%) with patchy consolidations, and 8 (16.3%) with small nodular opacities exhibited improvement after treatment. In contrast, no patients in the control group demonstrated significant improvements. No significant differences were observed in the improvement rates of patients with semiconfluent small opacities, PMF, and reticulonodular opacities between the two groups (Table 2).

In the control group, the area of PMF was enlarged in 4 patients, reticulonodular opacities were increased in 10 patients, whereas new PMF and reticulonodular opacities were observed in 7 and 4 patients, respectively. In contrast, in the observation group, the area of PMF was enlarged in only two patients, reticulonodular opacities were increased in only one patient, and new PMF was noted in three patients. No patients had new reticulonodular opacities. PMF and reticulonodular opacities were significantly worse in the control group than in the observation group.

**Table 2:** Changes in HRCT findings after treatment

HRCT features	Group	Not improved	Improved	χ^2^	*p*-value
Small nodular opacities	Control	24 (100)	0	4.401	0.047
	Observation	41 (83.7)	8 (16.3)		
GGO	Control	13 (100)	0	17.425	0.000
	Observation	8 (29.6)	19 (70.4)		
Semiconfluent small opacities	Control	4 (100)	0	0.431	1.000
	Observation	9 (90.0)	1 (10.0)		
PMF	Control	7 (100)	0	0.430	1.000
	Observation	16 (94.1)	1 (5.9)		
Reticulonodular opacities	Control	20 (100)	0	0.938	1.000
	Observation	42 (95.5)	2 (4.5)		
Patchy consolidation	Control	19 (100)	0	13.843	0.000
	Observation	16 (50.0)	16 (50.0)		

Data are presented as n (%)

*GGO* Ground-glass opacities, *HRCT* High-resolution computed tomography, *PMF* Progressive massive fibrosis

### Pulmonary function results

No significant differences were noted in FVC, FVC%, FEV_1_, FEV_1_%, or DLCO% between the two groups at follow-up (*p* > 0.05). However, changes from baseline to follow-up in all five lung function parameters were significantly different between the two groups (*p* < 0.001). The observation group exhibited a slight but significant increase in FVC and FVC% (*p* < 0.05), whereas the control group exhibited a significant decrease in all parameters (*p* < 0.001) (Table 3).

**Table 3:** Changes in pulmonary function data before and after treatment

Parameters	Group (*n*)	Before	After	t	*p*-value
FVC%	Control (26)	73.6 ± 21.2	67.9 ± 22.8	−3.805	0.001
	Observation (53)	71.6 ± 17.8	74.1 ± 20.1	2.625	0.011
FVC (mL)	Control (26)	3033.9 ± 896.7	2787.3 ± 939.5	−3.967	0.001
	Observation (53)	2933.0 ± 852.4	3025.5 ± 925.7	2.392	0.020
FEV_1_%	Control (26)	71.9 ± 23.2	64.1 ± 26.1	−5.084	0.000
	Observation (53)	66.7 ± 22.2	67.4 ± 23.7	0.791	0.433
FEV_1_ (mL)	Control (26)	2440.4 ± 769.9	2166.2 ± 859.4	−4.999	0.000
	Observation (53)	2263.2 ± 845.6	2280.6 ± 897.5	0.607	0.546
DLCO%	Control (23)	84.0 ± 22.0	74.0 ± 19.2	−4.219	0.000
	Observation (47)	76.2 ± 20.1	79.9 ± 21.3	1.526	0.134

*DLCO%* Diffusing capacity for carbon monoxide as a percentage of the predicted value, *FEV*_*1*_ Forced expiratory volume in one second, *FEV*_*1*_*%* percentage of the predicted FEV_1_, *FVC* Forced vital capacity, *FVC%* Percentage of the predicted FVC

**Fig. 2:**
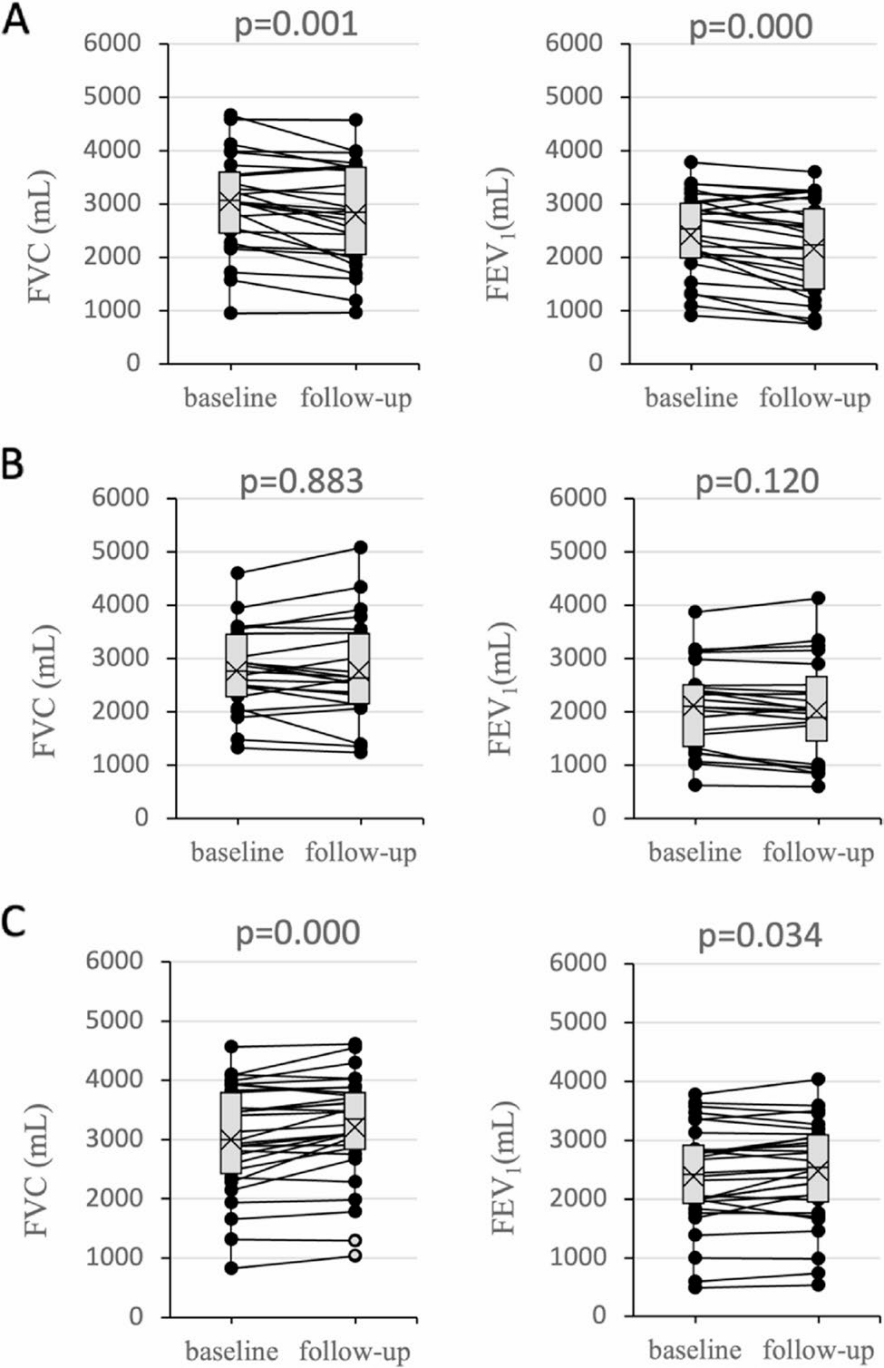
Changes in FVC and FEV_1_. **A** Control group; **B** ≤ 6 months of tetrandrine treatment subgroup; and **C** > 6 months of tetrandrine treatment subgroup

Further subgroup analysis was performed within the observation group based on tetrandrine treatment duration (≤ 6 months vs. > 6 months). At baseline, no significant differences were noted in any of the parameters between the two subgroups (*p* > 0.05). However, after 12 months, the increases in FVC, FVC%, FEV_1_, and FEV_1_% were significantly higher in the > 6 months subgroup than in the ≤ 6 months subgroup (*p* < 0.05), whereas changes in DLCO% were not significant between the two subgroups (*p* = 0.055). In the > 6 months subgroup, FVC and FEV_1_ significantly increased by 170.7236.1 mL and 80.3 ± 200.4 mL (*p* < 0.05), respectively, whereas they remained stable in the ≤ 6 months subgroup (*p* > 0.05). In contrast, FVC and FEV_1_ decreased significantly by 246.5 ± 316.9 mL and 274.2 ± 279.7 mL, respectively (*p* < 0.05), in the control group (Fig. 2).

### Adverse drug reactions

The reported side effects in the observation group included facial pigmentation (*n* = 15, 28.3%); transient skin itching, which was mild and resolved within 1 week (*n* = 5, 9.4%); mild liver function abnormalities not exceeding three times the upper normal limit, which returned to normal with hepatoprotective treatment (*n* = 5, 9.4%); transient diarrhea presenting as loose stools within 1 week of drug initiation, lasting a maximum of 2 days (*n* = 3, 5.7%); and mild appetite loss (*n* = 1, 1.9%). No significant adverse events were observed.

## Discussion

Silicosis-induced pulmonary fibrosis is progressive and irreversible [5, 19]. The standard management approach includes cessation of dust exposure and symptomatic treatment. However, artificial stone-associated silicosis differs from natural stone-related silicosis in that even a shorter duration of dust exposure can lead to a more aggressive disease, characterized by rapid progression on imaging and pulmonary function decline [13, 20]. For these patients, in addition to early detection, cessation of dust exposure, and symptomatic treatment, more effective strategies are urgently needed to control disease progression. Tetrandrine, a therapeutic agent for silicosis screened in China in the 1980 s, inhibits pulmonary fibrosis through multiple mechanisms in animal studies [21–24], making it a promising candidate for managing artificial stone-associated silicosis progression. The present study evaluated the efficacy of tetrandrine in slowing the progression of artificial stone-associated silicosis. The findings demonstrated that tetrandrine treatment in patients with artificial stone-related silicosis significantly improves lung function and HRCT imaging findings. A notable proportion of patients receiving tetrandrine exhibited reduced disease progression, highlighting the potential therapeutic benefits of tetrandrine in this specific population.

The findings of the present study indicated that after a 1-year follow-up, 49.1% of patients in the observation group exhibited improvement in chest HRCT findings compared with baseline. In contrast, no patient in the control group demonstrated such improvement, with a significant number of patients exhibiting a worsening condition. A similar pattern was observed in the pulmonary function results, with increases in FVC, FVC%, FEV_1_, FEV_1_%, and DLCO% in the observation group but a significant decline in all parameters in the control group. These results align with those of recent studies [25], although the imaging improvement rate in this study was higher than the 22.4% rate reported in a previous study on rapidly progressive silicosis treated with tetrandrine from the 1990 s [26]. The increased improvement rate in the observation group and the elevated deterioration rate in the control group in our study may be attributed to differences in patient populations. This is because our study focused on artificial stone-associated silicosis, which differs from natural stone-related cases examined in the previous studies.

In the present study, we specifically examined changes in different HRCT manifestations before and after tetrandrine treatment. The observation group exhibited significantly higher improvement rates for GGO, patchy consolidations, and small nodular opacities than the control group, which is consistent with the findings of a previous study [25]. GGO and patchy consolidations may be linked to extensive lipoprotein deposition in the lungs [27]. Animal studies have suggested that tetrandrine inhibits lipid metabolism, reducing periodic acid-Schiff and Sudan IV-positive deposits in the alveolar cavity of silicosis models [21], potentially contributing to significant disease control. However, in the present study, the improvement rates for reticulonodular opacities, semi-confluency of small opacities, and PMF did not differ significantly between the two groups. As PMF is histopathologically characterized by centrally hyalinized collagen fibrotic tissue [28], treatment with tetrandrine is unlikely to resolve these formations, which may partly explain this result. Nonetheless, given that the control group had an increased number of patients with worsening or new reticulonodular opacities and PMF, whereas most patients in the observation group maintained stability in these features, tetrandrine treatment still holds clinical value. Notably, in this study, we categorized loosely structured consolidations without well-defined boundaries as patchy consolidations rather than PMF. These “PMF-like” changes may not represent irreversible fibrotic lesions, potentially explaining their relatively better response to tetrandrine treatment.

Further subgroup analyses based on treatment duration revealed significantly greater improvement in HRCT findings and pulmonary function in patients treated for more than 6 months. Regression analysis also indicated a positive association between a longer treatment duration and improved HRCT findings. This suggests that over a 1-year period, patients receiving prolonged tetrandrine treatment experienced improved outcomes. To date, to the best of our knowledge, no study has directly compared the clinical effects of regular and irregular tetrandrine use. Some patients in this study did not strictly adhere to the regular treatment regimen owing to economic constraints, providing us with the opportunity to analyze the influence of treatment duration on disease control. These results suggest that the current tetrandrine protocol is an effective and tolerable treatment and that suboptimal dosing or irregular use leads to reduced efficacy.

This study has some limitations. First, as a retrospective study, missing clinical data and incomplete key examination results may have introduced selection bias. Second, the imbalance in the number of patients between the two groups may have affected the statistical power and precision of certain comparisons. Third, patients with better treatment responses were more likely to return for follow-up, potentially affecting the final conclusions. Fourth, adverse event data were collected based on patient-reported symptoms and auxiliary examinations, making a comprehensive assessment difficult. Finally, the observation period of this study spanned only one year, which is insufficient to demonstrate the long-term efficacy of tetrandrine.

To the best of our knowledge, this is the first study to analyze the effect of tetrandrine on different radiological patterns on HRCT and the impact of treatment duration on tetrandrine efficacy in patients with artificial stone-associated silicosis. This study provides valuable clinical insight into the types of radiological changes that may respond best to tetrandrine treatment, offering important information for clinical decision-making. As a complementary therapy to the current management strategies for artificial stone-associated silicosis patients, tetrandrine should be taken into consideration.

## Conclusion

This study demonstrated the significant therapeutic effects of tetrandrine in preventing the deterioration of pulmonary function and HRCT findings in patients with artificial stone-associated silicosis. The results suggest that tetrandrine may be a promising complementary management for these patients, offering hope for improved outcomes. However, to further confirm the efficiency of tetrandrine against artificial stone-associated silicosis, long-term, prospective, randomized controlled studies are warranted.

## Data Availability

All data relevant to the study are included in the article or uploaded as supplementary information.

## Abbreviations

BMI: Body mass index
DLCO%: Diffusing capacity for carbon monoxide as a percentage of the predicted value
FEV_1_: Forced expiratory volume in one second
FVC: Forced vital capacity
GGO: Ground–glass opacities
HRCT: High–resolution computed tomography
FEV_1_%: Percentage of the predicted value
FVC%: Percentage of the predicted value
RCS: Respirable crystalline silica
STROBE: Strengthening the Reporting of Observational Studies in Epidemiology
PMF: Progressive massive fibrosis

## References

[1] Leung CC, Yu ITS, Chen W. Silicosis. Lancet. 2012;379:2008–18. 10.1016/S0140-6736(12)60235-9. 22534002 10.1016/S0140-6736(12)60235-9

[2] León-Jiménez A, Hidalgo-Molina A, Conde-Sánchez MÁ, Pérez-Alonso A, Morales-Morales JM, García-Gámez EM, et al. Artificial stone silicosis: Rapid progression following exposure cessation. Chest. 2020;158:1060–8. 10.1016/j.chest.2020.03.026. 32563682 10.1016/j.chest.2020.03.026

[3] National Institute for Occupational Safety and Health. Health effects of occupational exposure to respirable crystalline silica. 2002. https://www.cdc.gov/niosh/docs/2002-129/default.html. Accessed 20 Mar 2025.

[4] Hoy RF, Chambers DC. Silica-related diseases in the modern world. Allergy. 2020;75:2805–17. 10.1111/all.14202. 31989662 10.1111/all.14202

[5] Handra CM, Gurzu IL, Chirila M, Ghita I. Silicosis: New challenges from an old inflammatory and fibrotic disease. Front Biosci (Landmark Ed). 2023;28:96. 10.31083/j.fbl2805096. 37258484 10.31083/j.fbl2805096

[6] Barnes H, Lam M, Tate MD, Hoy R. Toward targeted treatments for silicosis. Curr Opin Pulm Med. 2024;30:185–94. 10.1097/MCP.0000000000001020. 37851380 10.1097/MCP.0000000000001020

[7] Ophir N, Shai AB, Alkalay Y, Israeli S, Korenstein R, Kramer MR, et al. Artificial stone dust-induced functional and inflammatory abnormalities in exposed workers monitored quantitatively by biometrics. ERJ Open Res. 2016;2:00086–2015. 10.1183/23120541.00086-2015. 27730180 10.1183/23120541.00086-2015 PMC5005163

[8] Leso V, Fontana L, Romano R, Gervetti P, Iavicoli I. Artificial stone associated silicosis: a systematic review. Int J Environ Res Public Health. 2019;16:568. 10.3390/ijerph16040568. 30781462 10.3390/ijerph16040568 PMC6406954

[9] Mandler WK, Qi C, Qian Y. Hazardous dusts from the fabrication of countertop: A review. Arch Environ Occup Health. 2023;78:118–26. 10.1080/19338244.2022.2105287. 35912480 10.1080/19338244.2022.2105287 PMC9909587

[10] Requena-Mullor M, Alarcón-Rodríguez R, Parrón-Carreño T, Martínez-López JJ, Lozano-Paniagua D, Hernández AF. Association between crystalline silica dust exposure and silicosis development in artificial stone workers. Int J Environ Res Public Health. 2021;18:5625. 10.3390/ijerph18115625. 34070293 10.3390/ijerph18115625 PMC8197517

[11] Martínez-González D, Carballo-Menéndez M, Guzmán-Taveras R, Quero-Martínez A, Fernández-Tena A. Evaluating silicosis risk: assessing dust constitution and influence of water as a primary prevention measure in cutting and polishing of silica agglomerates, granite and marble. Environ Res. 2024;251:118773. 10.1016/j.envres.2024.118773. 38522742 10.1016/j.envres.2024.118773

[12] Chen CH, Tsai PJ, Chang WW, Chen CY, Chen CY, Yates D, et al. Dose-response relationship between lung function and chest imaging response to silica exposures in artificial stone manufacturing workers. Environ Health. 2024;23:25. 10.1186/s12940-024-01067-1. 38429786 10.1186/s12940-024-01067-1 PMC10908069

[13] Wu N, Xue C, Yu S, Ye Q. Artificial stone-associated silicosis in China: a prospective comparison with natural stone-associated silicosis. Respirology. 2020;25:518–24. 10.1111/resp.13744. 31828940 10.1111/resp.13744 PMC7187561

[14] Chan EWC, Wong SK, Chan HT. An overview on the chemistry, pharmacology and anticancer properties of tetrandrine and fangchinoline (alkaloids) from Stephania tetrandra roots. J Integr Med. 2021;19:311–6. 10.1016/j.joim.2021.01.001. 33583757 10.1016/j.joim.2021.01.001

[15] Bhagya N, Chandrashekar KR. Tetrandrine–A molecule of wide bioactivity. Phytochemistry. 2016;125:5–13. 10.1016/j.phytochem.2016.02.005. 26899361 10.1016/j.phytochem.2016.02.005

[16] Wang Y, Cheng B, Lin YJ, Wang R, Xuan J, Xu HM. Preliminary study on the effect and molecular mechanism of tetrandrine in alleviating pulmonary inflammation and fibrosis induced by silicon dioxide. Toxics. 2023;11:765. 10.3390/toxics11090765. 37755775 10.3390/toxics11090765 PMC10536946

[17] Jones CM, Pasricha SS, Heinze SB, MacDonald S. Silicosis in artificial stone workers: Spectrum of radiological high-resolution CT chest findings. J Med Imaging Radiat Oncol. 2020;64:241–9. 10.1111/1754-9485.13015. 32157793 10.1111/1754-9485.13015

[18] Hoy RF, Baird T, Hammerschlag G, Hart D, Johnson AR, King P, et al. Artificial stone-associated silicosis: A rapidly emerging occupational lung disease. Occup Environ Med. 2018;75:3–5. 10.1136/oemed-2017-104428. 28882991 10.1136/oemed-2017-104428

[19] Yang B, Liu X, Peng C, Meng X, Jia Q. Silicosis: From pathogenesis to therapeutics. Front Pharmacol. 2025;16:1516200. 10.3389/fphar.2025.1516200. 39944632 10.3389/fphar.2025.1516200 PMC11813918

[20] Quan H, Wu W, Yang G, Wu Y, Yang W, Min C, et al. Risk factors of silicosis progression: a retrospective cohort study in China. Front Med (Lausanne). 2022;9:832052. 10.3389/fmed.2022.832052. 35445039 10.3389/fmed.2022.832052 PMC9013759

[21] Jiang HX. Preliminary study of the mechanism of silicosis therapy by tetrandrine. Zhonghua Jie He He Hu Xi Xi Ji Bing Za Zhi. 1983;6:92–4. PMID: 6628134. 6628134

[22] Li QL. Autopsy and histochemical analysis: Report of a case of silicosis treated with tetrandrine. Zhonghua Jie He He Hu Xi Xi Ji Bing Za Zhi. 1982;5:243–4. PMID: 7172937. 7172937

[23] Song MY, Wang JX, Sun YL, Han ZF, Zhou YT, Liu Y, et al. Tetrandrine alleviates silicosis by inhibiting canonical and non-canonical NLRP3 inflammasome activation in lung macrophages. Acta Pharmacol Sin. 2022;43:1274–84. 10.1038/s41401-021-00693-6. 34417574 10.1038/s41401-021-00693-6 PMC9061833

[24] Ma R, Huang X, Sun D, Wang J, Xue C, Ye Q. Tetrandrine alleviates silica-induced pulmonary fibrosis through PI3K/AKT pathway: Network pharmacology investigation and experimental validation. Inflammation. 2024;47:1109–26. 10.1007/s10753-023-01964-6. 38265677 10.1007/s10753-023-01964-6

[25] Wu WH, Feng YH, Min CY, Zhou SW, Chen ZD, Huang LM, et al. Clinical efficacy of tetrandrine in artificial stone-associated silicosis: A retrospective cohort study. Front Med (Lausanne). 2023;10:1107967. 10.3389/fmed.2023.1107967. 36873890 10.3389/fmed.2023.1107967 PMC9981789

[26] Li DH. Task group of clinical evaluation on therapeutic effects of drug treatment for silicosis. Clinical trial and evaluation of treatment for silicosis. Chin J Ind Hyg Occup Dis. 1996;14:130–4.

[27] Marchiori E, Souza CA, Barbassa TG, Escuissato DL, Gasparetto EL, Souza AS Jr. Silicoproteinosis: high-resolution CT findings in 13 patients. AJR Am J Roentgenol. 2007;189:1402–6. 10.2214/AJR.07.2402. 18029877 10.2214/AJR.07.2402

[28] Chong S, Lee KS, Chung MJ, Han J, Kwon OJ, Kim TS. Pneumoconiosis: comparison of imaging and pathologic findings. RadioGraphics. 2006;26:59–77. 10.1148/rg.261055070. 16418244 10.1148/rg.261055070

